# Internal Hemipelvectomy for primary bone sarcomas using intraoperative patient specific instruments- the next step in limb salvage concept

**DOI:** 10.1186/s12891-022-05918-1

**Published:** 2022-11-24

**Authors:** Amit Benady, Yair Gortzak, Summer Sofer, Yuval Ran, Netta Rumack, Avital Elias, Ben Efrima, Eran Golden, Ortal Segal, Omri Merose, Amir Sternheim, Solomon Dadia

**Affiliations:** 1 Levin Center of Surgical Innovation and 3D Printing, Tel Aviv Sourasky Medical Center, Tel Aviv, Israel; 2 Sackler School of Medicine, Tel Aviv University, Tel Aviv, Israel; 3 Division of Orthopaedic Surgery, Tel Aviv Medical Center, Tel Aviv, Israel; 4 National Unit of Orthopedic Oncology, Tel Aviv Sourasky Medical Center, Tel Aviv, Israel; 5 The Military Track of Medicine, The Hebrew University-Hadassah Medical School, Jerusalem, Israel; 6 Office of the Deputy Medical Manager, Tel Aviv Sourasky Medical Center, Tel Aviv, Israel

**Keywords:** Pelvic bone tumors, Internal hemipelvectomy, 3D pre-operative planning, Patient specific instruments (PSI), Anatomical models

## Abstract

**Background:**

During pelvic Sarcoma resections, Surgeons often struggle to obtain negative margins while minimizing collateral damage and maintaining limb function. These complications are usually due to the complex anatomy of the pelvis. Here we present an accurate 3D surgical approach, including pre-operative printing of models and intraoperative patient-specific instruments (PSIs) for optimizing pelvic sarcoma resections.

**Methods:**

This single-center retrospective study (*N* = 11) presents surgical, functional, and oncological outcomes of patients (average age 14.6 +/− 7.6 years, 4 males) who underwent pelvic sarcoma resections using a 3D surgical approach between 2016 and 2021. All patients were followed up for at least 24 months (mean = 38.9 +/− 30.1 months).

**Results:**

Our results show promising surgical, oncological, and functional outcomes. Using a 3D approach, 90.9% had negative margins, and 63.6% did not require reconstruction surgery. The average estimated blood loss was 895.45 ± 540.12 cc, and the average surgery time was 3:38 ± 0.05 hours. Our results revealed no long-term complications. Three patients suffered from short-term complications of superficial wound infections. At 24 month follow up 72.7% of patients displayed no evidence of disease. The average Musculoskeletal Tumor Society (MSTS) score at 12 months was 22.81.

**Conclusion:**

3D technology enables improved accuracy in tumor resections, allowing for less invasive procedures and tailored reconstruction surgeries, potentially leading to better outcomes in function and morbidity. We believe that this approach will enhance treatments and ease prognosis for patients diagnosed with pelvic sarcoma and will become the standard of care in the future.

## Introduction

Sarcomas are malignant mesenchymal tumors that originate in the bone or soft tissue and account for around 1% of solid malignancies in adults and about 20% in children. While most sarcomas are soft tissue sarcomas, just over 10% are malignant tumors of the bone. Around 15% of all primary bone sarcomas involve the pelvic girdle [1, 2]. For these tumors, a margin-free surgical resection with or without (neo)-adjuvant treatment, depending on tumor grade, is the standard of care [3]. Due to the risk of local recurrence, a negative margin resection that involves the entire tumor mass and a margin of surrounding healthy tissue is necessary to provide adequate treatment. However, achieving sufficient resection margins in the pelvic region is complicated because of tumor size, proximity to critical anatomic structures, and the complex 3D pelvic anatomy [4]. Pelvic sarcomas often present late, and at the time of diagnosis they are relatively large [5]. Beyond this, the composition of the pelvis such as the proximity to surrounding organs and neurovascular structures makes resection of tumors in the pelvis a highly intricate process. When operating free hand, wound infections, blood loss, pelvic instability, and nerve or visceral damage are the main complications of internal hemipelvectomy [6]. Therefore, these resections are challenging even for experienced surgeons [7].

Following tumor resection, the main concern is local recurrence. In patients with intralesional or marginal margins, Sherman et al. showed local recurrence in 40% of patients; however, in cases with wide margins, recurrence occurred in only 9% of patients [8]. These results were supported by Farfalli et al., who demonstrated a rate of 30% local recurrence following primary bone sarcomas treated with limb salvage surgery [9]. Other common complications following internal hemipelvectomies are infections, bleeding, and injury to local structures such as nerve injuries in addition to bladder and bowel injuries. In the existing literature, the rate of postoperative complications varies from 20 to 60% with infection being the most common complication, followed by wound healing complications [10].

Previously, the mainstay of treatment for malignant pelvic tumors was external hemipelvectomy (hindquarter amputation). However, with the advancement of neoadjuvant therapies and surgical techniques, internal hemipelvectomy (limb salvage surgery) has become increasingly successful [11]. Still, even with these improvements, the prognosis for patients with primary bone sarcomas of the pelvis is considerably less favorable than for patients with primary bone sarcomas of the extremities both in terms of overall survival and in terms of local recurrence rates [12]. While the 5-year survival for localized extremity osteosarcomas approaches 70%, in pelvic osteosarcomas the 5-year survival is approximately 30% [13, 14].

Here we present a surgical method using three-dimensional (3D) modeling. This surgical technique reveals how intra-operative Patient Specific Instruments (PSIs) can improve the accuracy of pelvic sarcoma resections. By using more advanced procedure planning that eases navigation, 3D technology provides enhanced precision margins and optimizes efficiency. Additionally, intraoperative PSIs are a valuable guide for the surgeon in improving pelvic reconstruction methods, including biological and endoprosthetic reconstructions. The objective of this study was to evaluate and describe the oncological and functional outcomes after limb salvage resections while using a novel workflow that includes 3D pre-planning and printing customized surgical guides for intraoperative use.

## Methods

### Patients

This retrospective study presents the postoperative outcomes of a cohort of patients with a primary bone sarcoma of the pelvis. The study includes patients that were operated on with a 3D surgical workflow between December 2016 and October 2020 (*n* = 11, average age 14.6 +/− 7.6 years, 4 males). Pathological diagnoses included Ewing sarcoma (*n* = 9) and osteosarcoma (*n* = 2). All patients were followed up for at least 24 months (mean = 38.9 +/− 30.1 months) and were seen in the clinic according to protocol (every three months during the first two years post-surgery, every six months during years 3 to 5, and once yearly after that) to assess oncological and functional outcomes. The Musculoskeletal Tumor Society Scoring (MSTS 1993) system was used to assess the functional status of the patients at a one-year follow-up. Surgical resection margins were categorized as R0- negative margins above 2 mm, R1- negative margins between 0 and 2 mm, and R2- positive margins. This study was approved by the Tel Aviv Medical Center ethics committee (0174–18-TLV). (For full details, see Tables 1 and 2).

**Table 1:** Demographics, Diagnostic and Surgical Outcomes

Patient	Age	Gender	Diagnosis	Resection Type	Reconstruction	Months follow up	Surgery Time (Hours)	Estimated blood loss (cc)
1	13	F	OSA	Type I	Autograft	36.4	2:25	1000
2	13	F	Ewing	Type I/IV	None	43.4	4:52	1800
3	7	F	Ewing	Type I	None	50.4	3:59	300
4	8	M	Ewing	Type I	None	54.4	2:59	500
5	15	M	Ewing	Type I	None	59	3:50	750
6	17	F	Ewing	Type II/III	Hip Revisions Components	26	2:55	600
7	17	F	OSA	Type III	Allograft	55.7	6:35	2000
8	17	F	Ewing	Type II/III	None	30.8	3:56	750
9	21	M	Ewing	Type I/IV	Cement	51.5	1:51	500
10	15	F	Ewing	Type III	None	51.8	2:43	1000
11	23	M	Ewing	Type I	None	36.1	3:58	650

**Table 2:** Post-Operative Functional and Oncological Outcomes

Patient	Complications		Margins	Necrosis	Oncologic Events	Oncological Survival	MSTS
	Short Term	long term					
1	Superficial infection	None	R0	80	Local Recurrence	AWD	24
2	None	None	R0	95		NED	15
3	None	None	R0	98		NED	28
4	None	None	R0	100		NED	22
5	None	None	R1	100	Lung Metastasis	AWD	10
6	None	None	R0	66	Lung Metastasis	AWD	18
7	Superficial infection	None	R0	60		NED	26
8	None	None	R1	100		NED	26
9	None	None	R2	100		NED	24
10	Superficial infection	None	R0	98		NED	29
11	None	None	R0	100		NED	29

### Pre-operative planning and simulation

To begin, the surgeon provided a biomedical engineer with a computerized tomographic (CT) scan and a Magnetic Resonance Imaging (MRI) scan of the pelvis. The CT scan slices provided were 0.5–1 mm thick and defined the exact bone anatomy, while the MRI scan slices provided were 4 mm thick and defined the tumor and soft tissue borders. All the 2D images obtained from both modalities were imported into an FDA-approved image-processing software (Mimics®, Materialise, N.V. Leuven, Belgium, or Intellispace Portal V9 and V11, Philips Healthcare, Best, Netherlands). The images were merged and segmented to produce a 3D digital model of the precise bone anatomy upon which the exact tumor margins were superimposed. Following segmentation and approval by the surgical team, the model was exported as an STL file into an FDA-approved CAD software program (3-matic®, Materialise N.V.). These tasks were all performed in the hospital. Based on the digital 3D model, the surgical team determined the best surgical approach for the specific tumor and clinical scenario and defined the surgical borders for tumor resection.

### 3D printed patient specific instruments

**Fig. 1:**
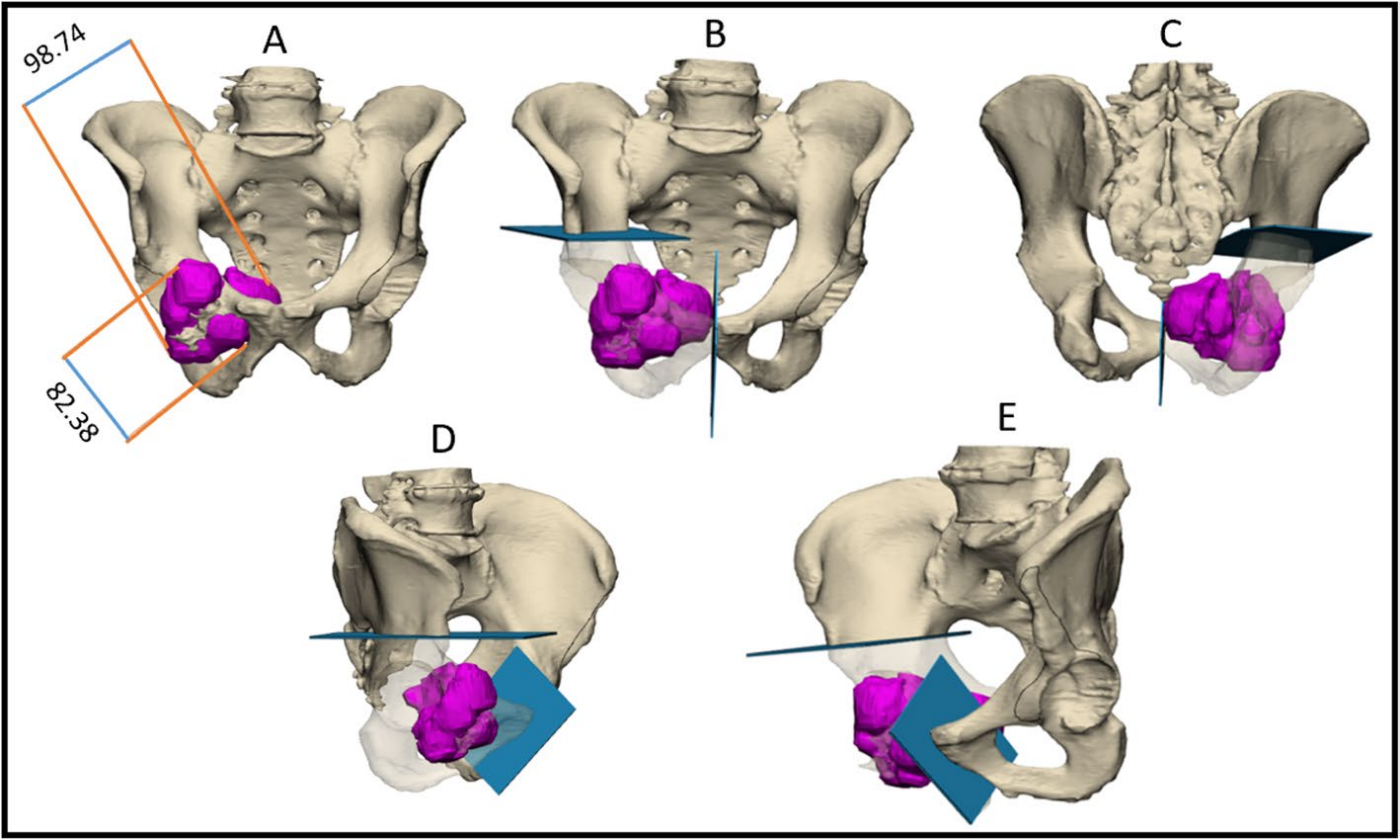
The precision of virtual 3D planning. A representative example of a digital 3D model of a tumor in the right ramus pubis. The tumor is demarcated in purple. Blue planes represent the pre-operative plan. *A. anterior* view, numbers show the tumor size (mm). B, C. Anterior and posterior views, respectively. D, E. Oblique views

**Fig. 2:**
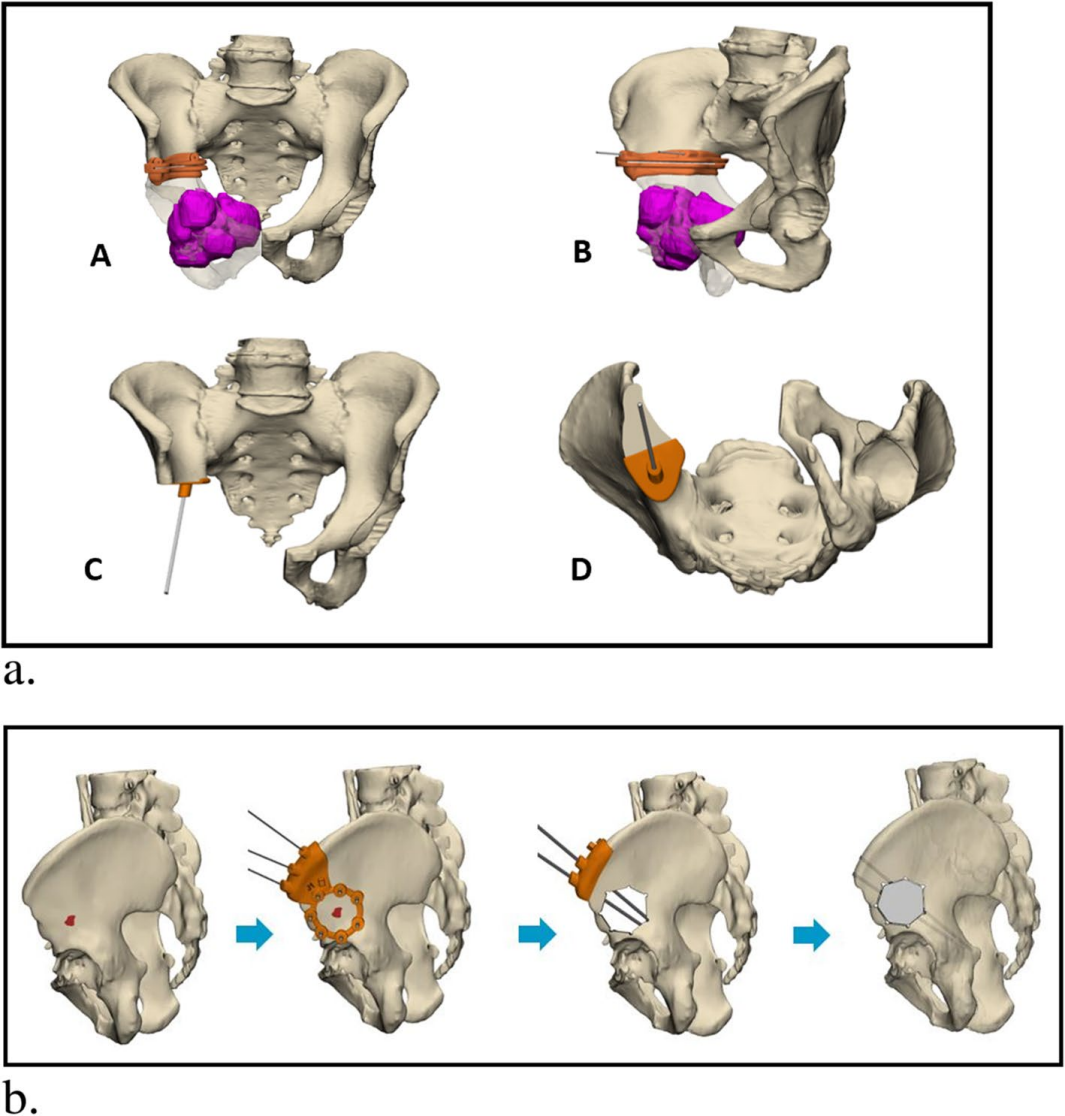
**a** A representative example of a digital 3D model of a tumor in the right ramus pubis. The tumor is demarcated in purple. Intraoperative PSIs are colored in orange. *A. anterior* view on an osteotomy guide. B. Oblique view of an osteotomy guide. C. Anterior view of a Lumic template. D. Inferior view of a Lumic template. **b**. Another example for the use of a PSI to resect a tumor with minimal healthy tissue loss. In this case, the bone defect was reconstructed with the allograft method

**Fig. 3:**
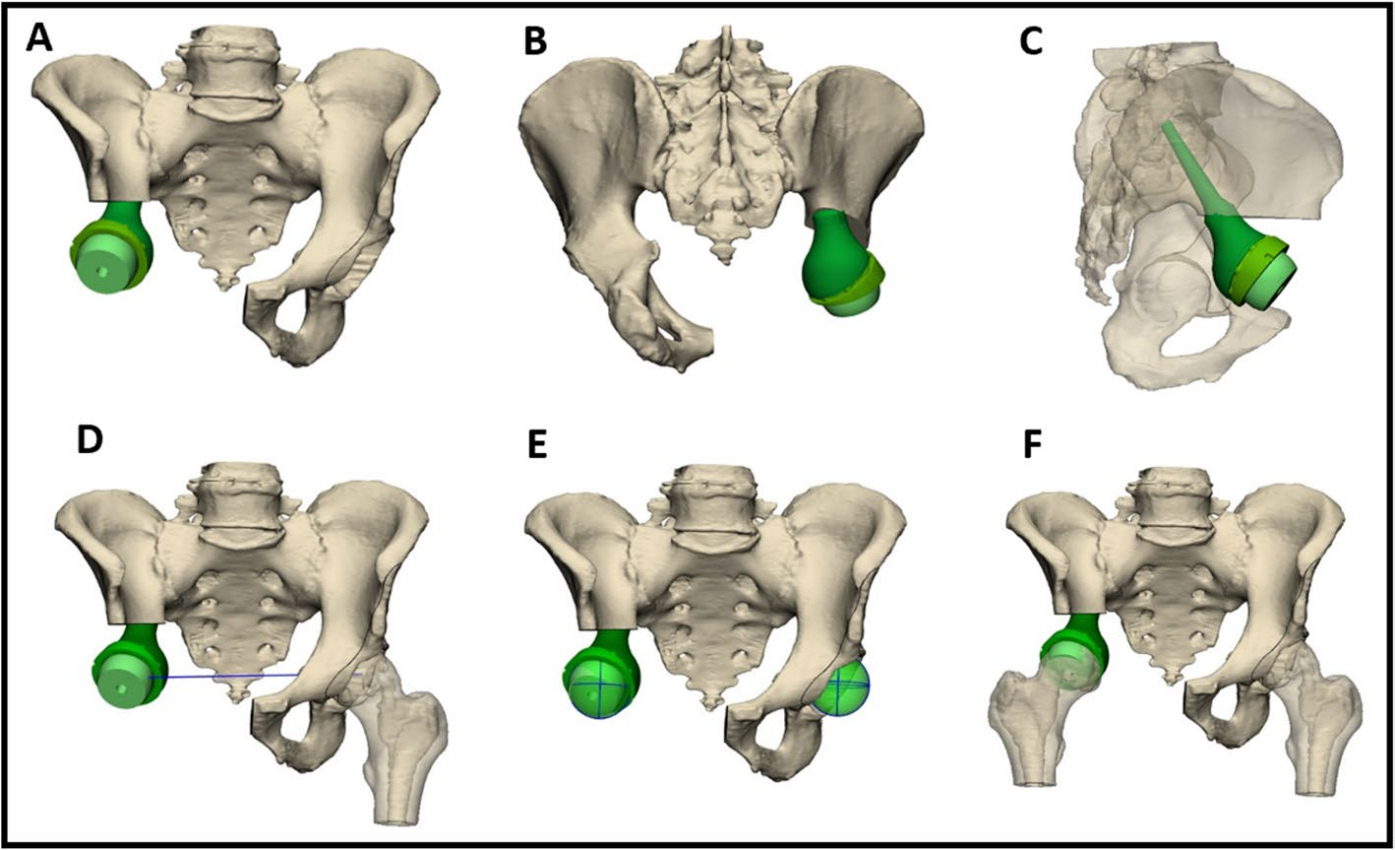
A representative example of a digital 3D model of a Lumic® reconstruction method. A main advantage of the 3D approach is the ability to conduct pre-operative digital simulation to design a tailored PSI and implant. The LUMiC® prosthesis is a modular device, built of a separate stem and acetabular cup and equipped with sawteeth at the junction to allow for rotational adjustment of cup position after implantation of the stem

**Fig. 4:**
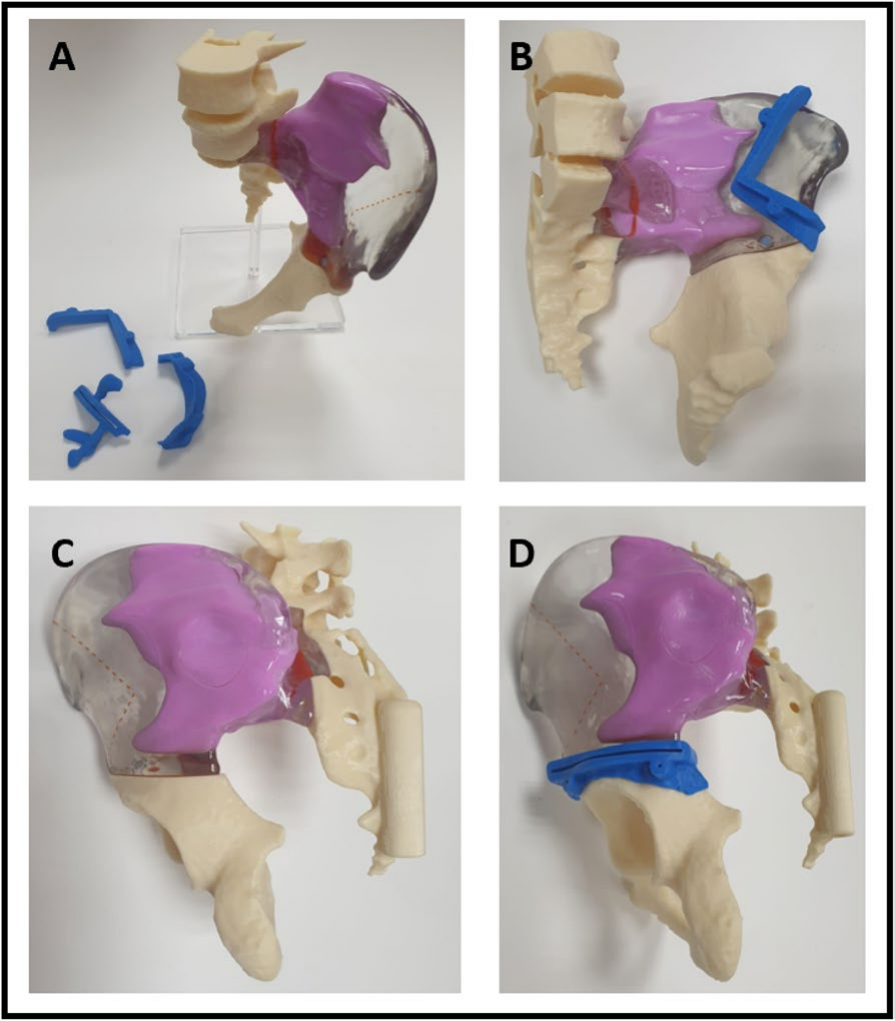
A physical 3D printed model in a 1:1 ratio of a tumor in the left iliac bone. The tumor is demarcated in purple. The iliac bone is transparent to show the extension of the tumor inside the bone. Blue parts are the PSI of the precise osteotomy

Medical designers helped mark the cutting planes for resection and a pre-surgical osteotomy plan was outlined (Fig. 1). After the surgeon was satisfied with the pre-surgical plan, a cutting Patient Specific Instrument (PSI) was then designed based on the desired cutting planes which would allow for accurate guidance during intraoperative osteotomies. Each PSI was planned with a unique footprint that followed the bone morphology of a specific patient to ensure complete placement. The PSI was a 1 mm thick slit at each cutting plane (Fig. 2). In cases where reconstruction was used, the implant was also planned using a 3D digital model. The main advantage of the 3D approach is the ability to conduct a pre-operative digital simulation to design a tailored PSI and implant (Fig. 3) [15]. After the engineer completed the virtual planning, it was reevaluated and approved by the surgeon. The cutting PSIs were then printed from biocompatible high-strength and thermal-resistant material (ULTEM™ 1010) by a Fused Deposition Modeling (FDM) printer (Fortus 450 mc, Stratasys, Eden Prairie, Minnesota; Rehovot, Israel). Finally, the PSIs were washed, double-packed, and underwent a standard autoclave sterilization process before they were brought to the surgical theatre. Additionally, a physical 3D model and PSI were printed in a 1:1 ratio so that the surgical plan could be validated in advance (Fig. 4).

## Results

In our cohort (*n* = 11), all patients underwent an internal hemipelvectomy. (For full details, including resection types see Tables 1 and 2). Our results showed that 90.0% of the cohort had negative margins. One patient underwent a planned positive margin resection (see elaboration in the discussion). Tumor necrosis was 90.6%. The average estimated blood loss was 895.45 ± 540.12 cc, and the average surgery time was 3:38 ± 0.05 hours. Reconstruction methods when needed, included autograft (*n* = 1), allograft (*n* = 1), PMMA (*n* = 1), and hip revision components (*n* = 1). Seven patients (63.6%) did not undergo reconstruction. Three patients suffered short-term complications including superficial wound infections (27.3%). There were no long-term complications. The average MSTS score at 12-month follow-up was 22.81. At 38.9 +/− 30.1 months follow-up, two patients presented with lung metastasis, and one had a local recurrence to the pelvis. Eight patients displayed no evidence of disease, (72.7% NED) and three patients were alive with disease (27.3%).

## Discussion

This study describes a 3D approach for oncologic resection of pelvic sarcomas. In pelvic tumor resections, there is a delicate balance between obtaining clear oncological margins, ensuring the entire tumor is resected, and avoiding resection of critical structures that will negatively influence future function. Although internal hemipelvectomies combined with chemotherapy and/or radiotherapy have improved oncological outcomes, functional outcomes are still poor compared to patients with bone sarcomas of the extremities [11]. In recent years, limb salvage surgeries for pelvic sarcomas have shown advancements in surgical techniques that have led to significantly improved outcomes while still maintaining minimal resection of healthy tissue. These methods include computed tomography (CT)-guided navigation with an O-arm [16, 17], optical navigation [18–21]and patient-specific instruments (PSIs) [22–25]. Intraoperative navigation and PSIs have been shown to improve accuracy compared to freehand tumor resection [7, 26, 27]. The contribution of these methods is particularly important when the tumor involves the pelvis. Previously, since the extension of the tumor in the bone is not visible, some bone osteotomies had to be made without any visual guidance. 3D digital pre-surgical planning and printed PSIs as intraoperative tools provide a means for defining accurate tumor margins and serve as a continuous guide during resection. When planning the procedure, we found that several factors can be implemented to improve the efficiency and efficacy of the process. For example, in designing a PSI, it is important for the surgeon to account for the specific blade that will be used with the oscillating saw during surgery and adapt the PSI accordingly. Additionally, to prevent overly strong vibrations, the cutting space where the blade is inserted should be 0.1–0.2 mm wider than the blade thickness and the oscillating saw’s range of motion. From our experience, the cutting PSI should be 1 cm in height and 2 mm thick for optimal stability. Overall, the PSI can be planned in such a manner that it allows for an accurate osteotomy in several planes in addition to improving surgical field exposure by retracting the soft tissue flap during the procedure [23].

Previous studies have demonstrated significant differences in local recurrence rates between intralesional, marginal, and wide margins in treating primary sarcomas. Therefore, treatment’s primary goal is to obtain clear oncological margins [9]. In a study conducted by Evrard et al., it was shown that when no guided system was used these tumors were associated with a local recurrence rate of 39% however when PSI guided resection was used, no patients presented with local tumor recurrence [27]. In our study, 90.9% of the patients had negative margins following resection using 3D technology and PSIs. Based on the surgeon’s pre-operative plan, one patient in our cohort deliberately underwent resection through the tumor with bone-planned positive margins. This patient received radiotherapy both pre- and post-surgery. In this situation, additional cryoablation and curettage were used on the remaining tumor within the supra-acetabular region of the Ileum. This approach allowed for the preservation of bone mass at crucial locations such as the acetabulum. In this unique case, the surgeons believed that preserving the acetabulum and preventing a significant disarticulation surgery governs the need for complete tumor resection with clear margins. From our experience, planned positive margins can be used only in cases of Ewing’s Sarcoma where the patients also receive radiotherapy, a treatment shown to be effective in Ewing’s sarcomas as these tumors are very sensitive to radiation therapy [28].Our extensive data shows that resection with planned positive margins in Ewing’s sarcoma does not present with a higher incidence of local recurrence or metastasis. Thus, in unique cases, we consider operating with planned positive margins when the functional outcomes are significantly better; this can be achieved by using PSIs to execute the exact pre-surgery plan.

Internal hemipelvectomy is a complex procedure than can be performed with curative intent, however, the procedure usually presents with many complications. Using 3D modeling and PSIs we can enhance oncological outcomes by minimizing certain complications such as blood loss and surgical times [29]. In a study conducted by Apffelstaedt JP et al., 32 internal hemipelvectomies were performed on an average of 7.5 hours, analysis showed that the average blood loss was 3.2 L [30]. In our cohort when using the 3D technologies, the average estimated blood loss was 927.27 ± 534.49 cc and the average surgery time was 3:35 ± 0:05 hours.

This technology impacts surgical outcomes and improves overall functionality, minimizing the need for reconstruction and improving allograft fit in cases where reconstruction is still necessary. A retrospective review was performed by Chao, A. H et al., where all patients who underwent internal hemipelvectomy between 1998 and 2011 in their institution were studied. They reported that out of 111 cases 54.1% of patients did not require reconstitution [31]. Salunke, A. A et al., evaluated the results of 23 patients who underwent internal hemipelvectomies and reported that 47.8% were without reconstruction [10]. Moreover, 3D planned margins allow the surgeon more freedom to obtain free supra-acetabular margins. This is a game changer, allowing what would previously result in a Type I-II resection, requiring reconstruction with an endoprosthetic implant, to be performed as a Type I resection instead. Reconstruction is optional in Type I resections and usually depends on the surgeons’ preference. Therefore, with accurate planning and a better understanding of the margins by using the 3D approach, 8 out of 11 patients (63.6%) in our cohort were able to undergo hip preservation surgery. Reconstruction leads to higher complication rates due to infection and loosening of hardware over time, avoiding reconstruction may lead to improved patient outcomes and less invasive surgeries [32, 33]. In a study of 270 patients undergoing internal hemipelvectomy, Angelini et al. demonstrated that the only significant factor contributing to deep infection was the reconstruction of the bony defect in the pelvic ring. They showed an infection rate of 15% for patients without reconstruction as opposed to 26% infection rate in patients that underwent reconstruction. When infection did occur, in almost half the patients it required the removal of implants and in some patients external hemipelvectomy was necessary to fully eradicate the infection [34]. Beyond this, even in cases where reconstruction is still required, using 3D planning and PSIs to guide allograft cuts works to improve the implant fit, leading to better biomechanics and enhanced functionality for patients [34]. Another advantage related to PSI includes direct visual control of the cutting depth, allowing for easy mobilization of the resected tumor specimen and increasing the safety of critical bone cuts. The major drawback of using the described 3D working flow is that each case requires a procedural team made up of surgeons, engineers, and medical designers (see methods for more details) to produce the desired outcome. We believe, that in the future technological advances and wider experience with this technology will make this limitation less pronounced and beyond this, the improved surgical and functional outcomes will validate the approach.

The high cost of implementing 3D technologies is also a significant limitation. 3D image workflow usually requires multiple imaging modalities (e.g., CT, MRI) that are not always available in every medical center due to their high cost. The specific equipment for each technology needs to be purchased and maintained. Furthermore, implementing these technologies requires coordinating a multidisciplinary team, as stated above. All the mentioned factors must be evaluated against the advantages of 3D technologies and their financial implications - reduced operation time, complications, and postoperative hospitalization. Several possible solutions have already been introduced to improve the cost effectiveness and increase the accessibility of 3D technologies by using Point-of-Care 3D printing centers, low-cost 3D printers, open-source software, and reusable materials [35, 36]. However, the cost-effectiveness of using PSI should be further evaluated and discussed concerning the economic aspect and not only the clinical/ medical part. Another limitation to this study is that it is a retrospective study. Additionally, this study includes a heterogeneous group of patients with differences in tumor characteristics such as size, location, and stage. To overcome these limitations, in the future there is a need for a prospective, multicenter study that includes a larger study group where patients are stratified according to their tumor subclass and the surgery performed.

In conclusion, despite the study’s limitations, which included a relatively small group and a broad variety of tumor features, we observed that the 3D technology plays a role in improving both the oncological and functional outcomes for patients with pelvic sarcomas. The use of 3D technology has long been recognized to result in more accurate bone resections, possibly resulting in fewer local recurrences and less invasive resections that may not require reconstruction, implying better functional outcomes. We expect that this method will become the standard of care in the future, resulting in enhanced tailored surgical treatments for orthopedic oncologic patients.

## Data Availability

All data generated or analyzed during this study are included in this published article (and its supplementary information files).

## Abbreviations

MSTS: Musculoskeletal Tumor Society Scoring
CT: Computed Tomography
MRI: Magnetic Resonance Imaging
STL: Standard Triangle Language
FDM: Fused Deposition Modeling
3D: Three Dimensional
PSI: Patient Specific Instrument
PMMA: Polymethyl Methacrylate
AWED: Alive with Evidence of Disease
NED: No Evidence of Disease
DOD: Dead of Disease
